# Correction and integration of solid-angle data from the azimuthally resolving 2D detector at ID06-LVP, ESRF

**DOI:** 10.1107/S1600577523008020

**Published:** 2023-10-17

**Authors:** Wilson A. Crichton, Jérôme Kieffer, Pierre Wattecamps, Valentin Valls, Gilles Berruyer, Marie Ruat, Vincent Favre-Nicolin

**Affiliations:** a ESRF – The European Synchrotron, 71 Avenue des Martyrs, Grenoble, Rhône-Alpes, France; RIKEN SPring-8 Center, Japan

**Keywords:** diffraction, detector, beamline, high pressure, extreme conditions

## Abstract

The unique diffraction geometry of ESRF beamline ID06-LVP offers continuous static 2D or azimuthally resolving data collections over all accessible solid angles available to the tooling geometry. The system is built around a rotating custom-built Pilatus3 CdTe 900k-W detector from Dectris, in a configuration equivalent to three butted 300k devices. As a non-standard geometry, here the method of alignment, correction and subsequent integration for any data collected over all solid angles accessible, or over any azimuthal range contained therein, are provided and illustrated by parameterizing and extending existing *pyFAI* routines. At 1° integrated intervals, and typical distances (2.0 m), the system covers an area of near 2.5 m^2^ (100 Mpx square equivalent), to 0.65 Å resolution, at 53 keV from a total dataset of some 312 Mpx. Standard FWHMs of SRM660a LaB_6_ vary from 0.005° to 0.01°, depending on beam size, energy and sample dimensions, and are sampled at an elevated rate. The azimuthal range per static frame ranges from <20° to ∼1° over the full range of the detector surface. A full 2θ–intensity data collection at static azimuth takes 1–3 s typically, and can be reduced to ms^−1^ rates for measurements requiring time-rate determination. A full solid-angle collection can be completed in a minute. Sample detector distances are accessible from 1.6 m to 4.0 m.

## Abstract 2

Corrections that allow for high-precision integration of part or full solid-angle datasets from the long-aspect-ratio Pilatus 900k-W device at ESRF beamline ID06-LVP are presented.

## 1. Angle-dispersive data collection from the large-volume device

One of the goals of ID06-LVP was to establish data collection specifically in angle-dispersive geometry with a monochromatic X-ray beam. This is most unusual for beamlines for large-volume-press devices, as access is somewhat limited by the geometry and construction of the tooling, and by the materials involved. It has been furthermore hampered by the generally small surface areas of detectors available, until recently, compared with the necessity to keep these independent of press movements and, thus, at longer sample–detector distances. Such setups, then, require high-energy work in order to access reasonable real-space resolutions. This is contrary to the efficiency of generally available detectors, of geometrical rejection setups, and it degrades the highest Δ*d*/*d* resolutions that elevated energies provide. In early testing, this was achieved by step-scanning a scintillation counter in a composite chord of linear translation and a rotation stage to recover a 2θ-orthogonal incidence angle. This was advantageous in terms of ultimate resolution as gauge volumes provided by the receiving and detector slit-pairs were, generally, significantly smaller than sample diameters at interesting wavelength and 2θ combinations. This situation provided a good level of background rejection from the multitude of potential scatterers that compose a large-volume assembly (*e.g.* Guignard & Crichton, 2014 ▸; Bernal *et al.*, 2014 ▸; Crichton *et al.*, 2016 ▸). Such a setup, at long distance, also has high inherent resolution, but with the evident downside that such resolutions require sampling at compatible rates. Data collections were, therefore, time-consuming (hours) and the whole system tedious to align in spite of the robustness of the basic unit and its simplicity (no more complicated than a KF-40 vacuum tube with two sets of vacuum-compatible JJ X-ray slits at either end, with coaxial Tl:NaI scintillator detector). It also allowed for collection in the horizontal plane only, which, while useful for two-stage 6/8 operation, did not allow for azimuthal (χ) resolution of diffraction peaks over solid angle, nor for static collections about the vertical fan of 6/6 and one-stage deformation assemblies.

The first significant step in two-dimensional resolution of 2θ–intensity data was the introduction of a linear pixelated detector, with solid-angle information resolved by rotating this detector about a near-beam axis (see, for example, Guignard & Crichton, 2015 ▸). As an industrial and high-energy-efficient device, the Detection Technologies X-series device was selected. Typically used for X-ray video investigation (baggage, tyre inspection, *etc.*), it also afforded continuous collection, with selectable punctual TIFF output of a time series of lines as a single image from delivered control code. Thus, with a suitable total exposure time selected and an internal 10 Hz frame rate, full solid-angle data collection could be collected in a, motor-compatible, minute and required only a TTL trigger to initiate. This negated any requirement for a continuous collection mechanism with synchronized motors and integration into ESRF data collection policies. A typical data collection, *viz.* at 1 image revolution^−1^, consisted of some 512 lines (mod32) of azimuthal information and was significantly better resolving than other similar devices. Related data collection strategies were also being conducted elsewhere with 1D detection that also claimed increased resolution over flat-plate equivalents for diamond-anvil-cell collections (Fisch *et al.*, 2015 ▸). This was not without its drawbacks; prime here was the data being native in (χ, 2θ)pixel (px) space, *i.e.* polar, rather than in Cartesian (2θ, 2θ)-equivalent px. They also did not extend to 0° 2θ, so no unique solution can be found for beam-centre and distance calibration by typical methods (there is no ellipse to fit). Indeed, despite these 2D data being instantly inspected, they were not directly fittable with most diffraction-based analyses as a 2D dataset, as this still requires pixel-to-2θ integration, which suffers from the same issue. Versions 16 *et seq* of *Fit2D* (Hammersley, 2016 ▸) incorporated new developments to deal with these issues. These included polar-specific calibration routines that could estimate distance independent from beam centre, and correct for beam and rotation axis offsets, in addition to the usual tilt and rotation corrections shared with conventional flat-plate Cartesian collections. This solution to 2D detection, and correction, was quite unique and offered a significant gain in time resolution; from a few hours to, typically, 3.2 s (32 lines at 10 Hz, 32× rebinned) exposure per static full-range 1D 2θ-I collections. It also gave access to 360° of solid angle over an area that was not accessible to standard flat-plate detectors; thus, a whole range of kinetic studies, rapid surveys of *p*,*T*-space for phase diagrams and deformation experiments were possible, at much reduced anticipated cost and at high efficiency, as all pixels were used all of the time – irrespective of the geometry imposed by the tooling used. However, in using the 1D detector, the high inherent spatial and diffraction resolution of the 0D setup was lost and required mitigation, largely with advanced amorphous ceramics. Nonetheless, the 1D detector ran for nearly a decade and amply demonstrated the proposition to the next upgrade, *i.e.* to a Dectris-based device.

### 1.1. Description of the setup

The highest operable energy at ID06-LVP, with the Si111 monochromator, is ∼53 keV. This offers the narrowest fan of real-space diffraction data and the highest penetrating power. The narrower fan affords substantial engineering solutions to anvil support required in high-stress regimes, *e.g.* for 6/6 deformation at lower mantle pressures or for assemblies with limited opening (Drickamer at ψ ± 13.5°). The wedges that support the primary anvil stage determine the maximum fan angle, though the second-stage assembly and beam-axial, ω, rotation of the press will further modify this (Fig. 1 ▸). The detector must be efficient in all of the following second-stage operation modes:

(i) 6/8 Carbide anvils; static position, detection via horizontal gap (general use).

(ii) 6/8 Diamond anvils; solid-angle detection through gap and anvils (UHP, texture, single-crystal samples).

(iii) 6/6 Carbide; static position, detection via vertical gap (high-throughput surveys: low loads, reasonable volumes, easy assembly).

(iv) 6/6 Diamond; azimuthal resolution for deformation.

(v) Drickamer; solid-angle collection to UHP, or high strains.

As the projected anvil gap of a 6/8 carbide assembly, to typical working distances, is of the order of ±10–15 mm about the horizontal plane, a long-aspect-ratio detector is apt. If our goal is to achieve real-space resolutions of 0.9 Å at our lower energy limit (at ∼33 keV) and ∼0.6 Å at our highest, from our maximum opening angles, then these fix our desired detector dimensions, which may be modified by some modest radial detector displacement (we are rarely interested in *d* longer than 12 Å). The space vacated about 0° is then used to mount a beam-axial camera, for direct WYSIWYG imaging and diffraction of the sample. There are three choices to fill the solid angle: horizontal translation of a vertical detector, vertical translation of a horizontal detector, or rotation of a long-radius device (Fig. 2 ▸). At any incremental position, only the latter can collect full-range 2θ–intensity images and retain time resolution. Combining ω rotation with a short working distance and our highest energy, data collections are available to 22.5° 2θ or *d* = 0.6 Å with the 900k-W detector (4439 × 195 px @ 0.172 mm pitch = 763.5 × 33.5 mm) for static azimuths. Further details on the detector characteristics are available from the manufacturer’s website (https://media.dectris.com/220930-Technical_Specification-DECTRIS_PILATUS3_X_CdTe_2M.pdf).

The increase in static dimensionality of the 900k-W device introduced some significant geometrical issues; prime among these was a quality-of-life effect: stacking 1D data – even unintegrated – allows the user to directly inspect data collections live, through simple continuous stacking of raw detector output. We cannot simply stack adjacent 2D images to produce a time (*etc.*) series output of the 2D result: we must integrate to 1D patterns first. This is trivial, but it changes the workflow and necessitates an efficient, live, monitoring tool. The beamline provides this through 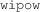 (of VF-N). Considering solid-angle data, at low scattering angles each static frame shares azimuthal contributions from the adjacent 15, or more, frames, but at highest angles from potentially only the next frames. Clearly, then, tilt and offset corrections are required *before* bi-dimensional intensity estimation per (2θ, χ) bin. When the native single image increased from only 1D binning (1539 px × 512 lines) without necessity for intermediate frame integration and correction to 900k px × 360 frames (with significantly higher bit depth) requiring correction *before* binning, a much more significant computational task is required to produce a fittable dataset. On the other hand, for static time-series collections, given that native Dectris images are Cartesian irrespective of the detector orientation in space, it remains a planar detector and can be integrated directly with standard 2D software; *e.g.* *Fit2D*, *pyFAI* (Ashiotis *et al.*, 2015 ▸), *Dioptas* (Prescher & Prakapenka, 2015 ▸), *etc*. However, the results obtained will not be generally compatible with the motor positions required to correct the resulting tilts, rotations, PONIs, *etc.*, as any tilts, or 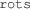 , will be defined about the beam centre, or point of normal incidence, rather than about the rotation centre. As bi-dimensional binning and integration (regrouping) will take minutes to complete, an on-the-fly precis of the current solid-angle dataset is readily available by accumulating near-beam-height regions of interest from adjacent frames in order to construct a direct polar image for inspection, or through integrating a region of interest over all the original images. This is of particular interest to stress–strain deformation experiments, which, due to practicalities in data collection rates, simply have no time for inspecting full integrations.

## 2. Preliminary studies

Upon receipt of the delivered detector, a series of site acceptance tests were undertaken; part of this was the estimation of the distortions of the pixel array. For details of this work, see https://www.silx.org/doc/pyFAI/dev/usage/tutorial/Detector/Pilatus_Calibration/Pilatus900kw-ID06.html.

## 3. Typical workflow

For alignment, once the standard (SRM660a) LaB_6_ (0.8/1.0 mm, vertically mounted capillary) has been located at the rotation centre of the press, pixel positions of low-angle diffraction peaks are matched at ordinal positions by displacing the detector vertically and horizontally, at a sample–detector distance estimated to be the most useful for the experiment. Using the lowest-angle peaks, we inspect and minimize the rotation-centre beam-position offset to better than 1 px. We are unable to correct for tilt and rotation mechanically, as an aid to keeping construction of the detector table manageable. The supporting granite is aligned near co-axially with the average monochromatic beam position and any remaining tilts are of the order of a few mrad about the ordinal axes. Should this functionality be available, then, following offset correction, high-angle peaks should be used for tilt correction.

Thereafter, MUSST-controlled detector rotation will be launched and will trigger data collections over the integrating period, typically 1°, of length 167–1667 ms (motor-compatible range) over the full revolution. The IcePAP motor controller (Janvier *et al.*, 2013 ▸) will be programmed so that the correct number of total images, over the total angular range and the exposure time per image, are all satisfied within valid limits. The detector will be rotating at full calculated speeds over the course of the integration time for all steps. A TTL signal provides control of both fast shutter, if used, and detector timing. Encoder positions or step counts via a P201 counting card will provide trigger points for data collection.

At completion, the data collected at χ = 270° are calibrated manually using *pyFAI-calib2* (Kieffer *et al.*, 2020 ▸), Fig. 3 ▸, with the returned PONI file serving as the initial parameters for full correction.

It is evident that a suite of 360 such calibrations could indeed be estimated and they will provide (usually) rather good estimations of the pixel–2θ calibration required, per azimuth. However, that work is not only tedious but it also fails to reflect the mechanics of the device, as this construction cannot be rotated: 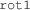 and 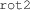 are defined relative to the pixel matrix, not the rotation axis. Such treatment cannot furthermore take into account the significant quantity of data on adjacent frames that contribute to that same azimuthal sector, especially if the data are further masked to include only ±0.5° of the sector about the nominal azimuthal position for the images (which should be the case). Furthermore, the resulting integrated sections also do not necessarily have the same 2θ range and, consequently, step, further complicating matters and eliminating other computation short-cuts, such as look-up pixel maps in 2θ–azimuth space.

### 3.1. Data correction

A *Jupyter Notebook*-based Python script (see example in Section S1 of the supporting information) has been prepared around the *pyfai-multigeometry* module, with necessary input from *single.geometry_refinement* at initial stages and follows the general goniometer calibration scheme illustrated by Kieffer *et al.* (2020 ▸).

Using the frame collected about 270°, an initial assessment of fit parameters and control points are made at all azimuthal positions, with the χ = 270° data returned for inspection (Fig. 4 ▸). A first round of refinement is then made at an assumed fixed orthogonal detector location through refinement of distance and beam centre only (Fig. 5 ▸) using the static calibration as initial. At this stage it is clear that there is a periodic behaviour in the distance returned, that is cyclic and is in phase with the estimation of the 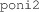 parameter. The 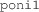 parameter does not appear, fortuitously, very significantly by any azimuthal effect in any of these cases.

The next stage updates the selection of control points, based on the returned PONI information, and the new parameter sets are introduced to 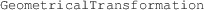 , where definitions of parameters used by 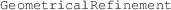 are described. These additional parameters are based on the rotation centre ( 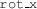 , 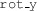 ) and how this affects PONI(χ) via regular rotation matrices; such that the beam centre precesses as

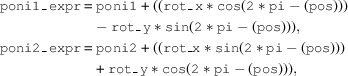

and the rotations about principle axes as

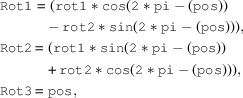

where 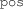 is the azimuthal position, χ, expressed in radians. A constrained refinement of all expressions and terms results in a root mean square value of 2 × 10^−9^ to 8 × 10^−10^ for a reduced dataset (five decades improved over initial calibrations), as shown in Fig. 6 ▸.

This information, together with the detector geometry, is sufficient to describe the location of idealized standard reference material (SRM) peak locations on the detector at any position, to further improve control point collection (with or without further extraction for problematic frames) and for final unconstrained refinement of all parameters over all frames.

From the output, it is clear that there is significant correlation and periodic behaviour, and that we are at the limit of pixelation in the results, *e.g.* in the envelope depicted by 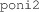 and 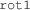 , Fig. 5 ▸, that correlate with variation in distance estimates per χ. We anticipate cyclic behaviour, and correlation between certain parameters is expected: the distance between the beam centre and the rotation centre (radius) is decomposed into its components (effectively 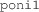 and 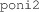 ) as a function of azimuth, as is the tilt, giving the oscillations in 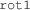 and 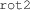 , with phase shift of π/2. The distance is not expected to vary, neither between frames nor with azimuth, unlike the forced-orthogonal case of distance in Fig. 5 ▸.

At this stage the multigeometry is defined and written in JSON. It is sufficient to set limits to the multigeometry integrator and initiate it. On a standard Dell laptop it takes ∼2 min for 360 azimuthal positions and 5000 radial bins. The data can be integrated at a much higher rate if necessary: 195 vertical pixels cover ∼1° of azimuth at longest radius. A comparison between the reproduced standard diffraction line positions and the detector slit gaps indicates the magnitude of the distortion (Fig. 7 ▸).

With three times oversampling, the 1D rebin is handled by 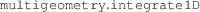 in 3 min, with some 12–14k intensity bins (see Fig. 8 ▸).

As an extra indicator of potential issues in the fitting, the full width at half-maximum (FWHM) is estimated over all peaks (Fig. 9 ▸), here at *E* = 52.7 keV and detx = 2150 mm, after having followed the same calibration as above. Misfits in rotation centre and beam offset would manifest in low angle broadening, and those due to tilt ( 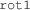 , 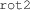 ) with higher angles. The slight increase observed is commensurate with a flat plate ∼2 m distant. For data with a higher 2θ range, it may be useful (for .par file construction) to fit Caglioti parameters (Caglioti *et al.*, 1958 ▸), Fig. 9 ▸.

Data processing of subsequent data collections forego any calibration and use of the JSON describing the experiment, coupled with 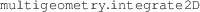 and 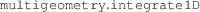 as required by the range in azimuth or expected use. The JSON can also be used to calculate independent PONIs at any given angle, or over any range; for example,

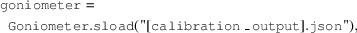




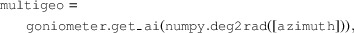

which returns the azimuthal integrator for use in single or batch instances of *pyFAI-integrate*.

## 4. Backwards compatibility

The data correction framework illustrated above is compatible with detector calibrations using the previous Detection Technology (DT) device. The most significant difference to the coding is the extraction of azimuthal data from the single dark-subtracted image output per full rotation and lack of a specific detector definition. The latter is generated with initial goniometer calibration in *pyfai-calib2*. Extraction of an individual row from the single image can be accomplished (for example see Section S2 of the supporting information). This will also ensure that the data can be used for control point extraction without any further modification of the script. In doing so, the vertical pixel size will be halved in the detector definition (in the DT definition it is arbitrarily set at [py] = 0.2 mm for square pixels). It then only remains to alter the 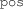 definition with respect to the row number in the original image. One may also have to alter the fit limits, as the origins of the DT detector and the Pilatus device are at opposite ends of the radius for the same azimuthal positions.

## 5. Further developments

The limits of correction can clearly be extended to the location of the detector with respect to the detector arm, *e.g.* if the detector surface is not parallel to the plane that the rotation describes. These ‘internal’ corrections will produce a conical integrated surface ( 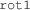 ), reduced azimuthal range per frame ( 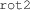 ) and compound precession about the rotation axis ( 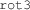 ). They are identifiable through estimates of pixel foreshortening and inexact estimations of tilt and rotation of the normal to the rotation axis. Such corrections are not required with this construction, but are included for completeness and will require a nesting of χ-independent PONI parameters within these χ-dependent parameters illustrated here. Further refinements can be added to the basic *Notebook* illustrated here: signal separation, for background removal, or inpainting detector chip gaps, for instance.

## Supplementary Material

Supporting information. DOI: 10.1107/S1600577523008020/yi5142sup1.pdf

Supporting information. DOI: 10.1107/S1600577523008020/yi5142sup2.html

Raw data, script & output.: https://zenodo.org/records/7957395

## Figures and Tables

**Figure 1:**
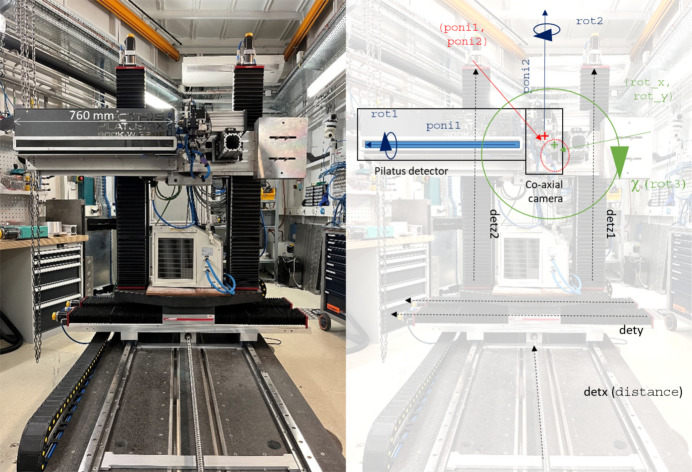
The left-hand image shows the downstream view of the detector table, with a key shown in the right-hand image. The Pilatus is mounted at χ = 270°. The beam centre is indicated in red and located at ( 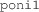 , 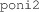 ), which are here modified by the rotation matrix. The axis directions and rotation about these are indicated ( 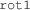 and 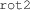 ); these are aligned with the pixel array and affected here by the rotation matrix. Rotation is provided by an azimuthal motor acting in a counter-clockwise direction (green) and about the axis, which intersects the distance plane at ( 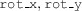 ) from the beam centre. The detector is movable in distance by detx, by coupled detz1 and detz2 tables (vertical) and by a pair of translations in the horizontal direction, only one of which is motorized (dety).

**Figure 2:**
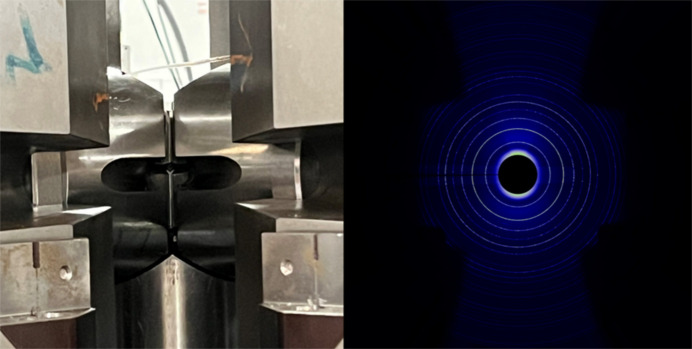
The image on the left, with open tooling and the bottom anvil in the reset position (fully extended at about −1.5 mm from the centre), shows the view upstream of the primary and wedge cut-outs, here with a carbide second-compression stage in 6/8 geometry. The maximum extent of the solid angle visible is limited by the anvil gap and the cut-out half-angle (13.5°) and ω rotation, to a maximum of ∼22°, in the horizontal plane. The right-hand panel shows a Cartesian reconstruction of a polar collection of SRM660a LaB_6_ (at 53 keV) in 6/6 geometry, showing the increased solid-angle range of collections possible. The limit is the wedges, except in sub-vertical azimuths, where the detector length constrains maximum 2θ. The off-ordinal accessibility is increased by ω rotation.

**Figure 3:**
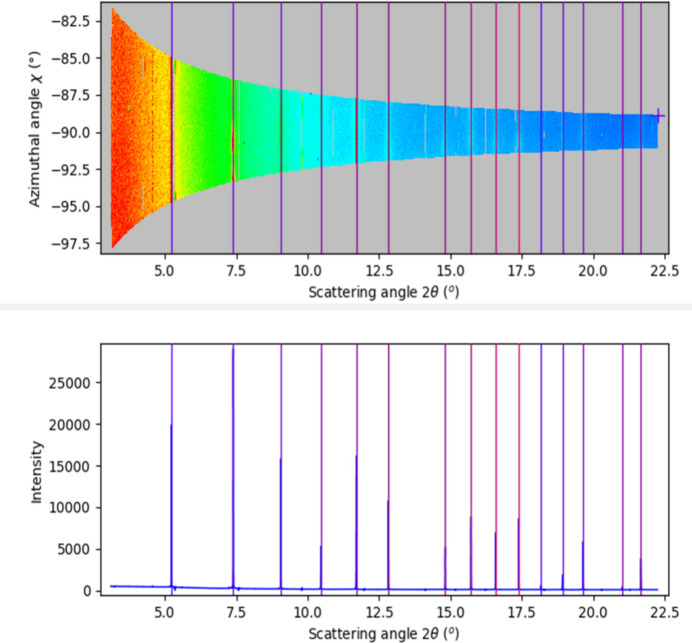
Treatment of static frames at 270° azimuth provides the initial input to the correction script, here with *pyFAI-calib2*. Shown here are the resulting 2D (top) and 1D (bottom) integrations. Notice the azimuthal range per frame.

**Figure 4:**
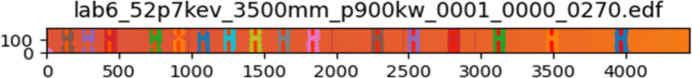
First extraction of control points from initial 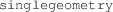 recalibration, based on the initial *pyfai-calib2* result, at *E* = 52.7 keV and detx = 3500 mm.

**Figure 5:**
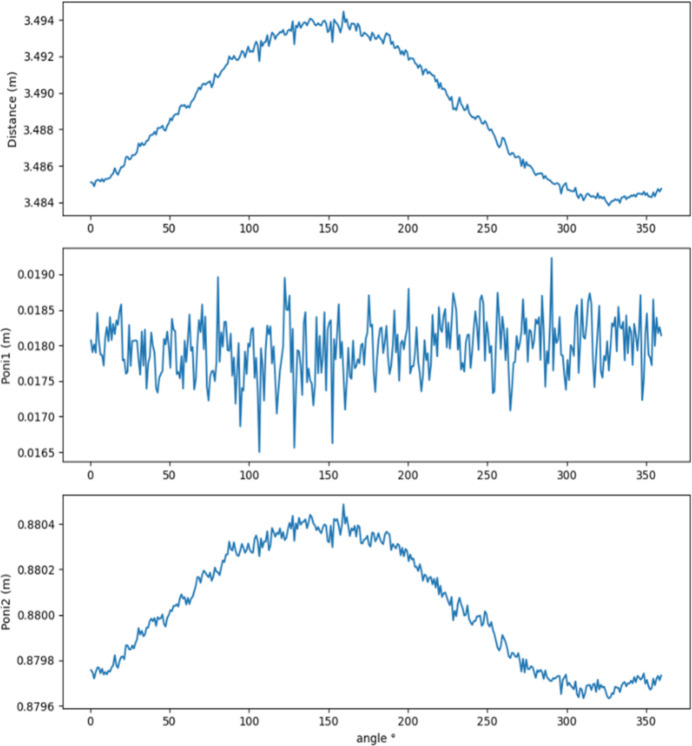
Results of the first round of recalibration, with refinement of 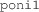 , 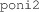 and distance only (*i.e.* assuming an orthogonal detector). Note the cyclic behaviour and high degree of correlation between resulting parameters per azimuth, where 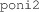 and distance appear to correlate.

**Figure 6:**
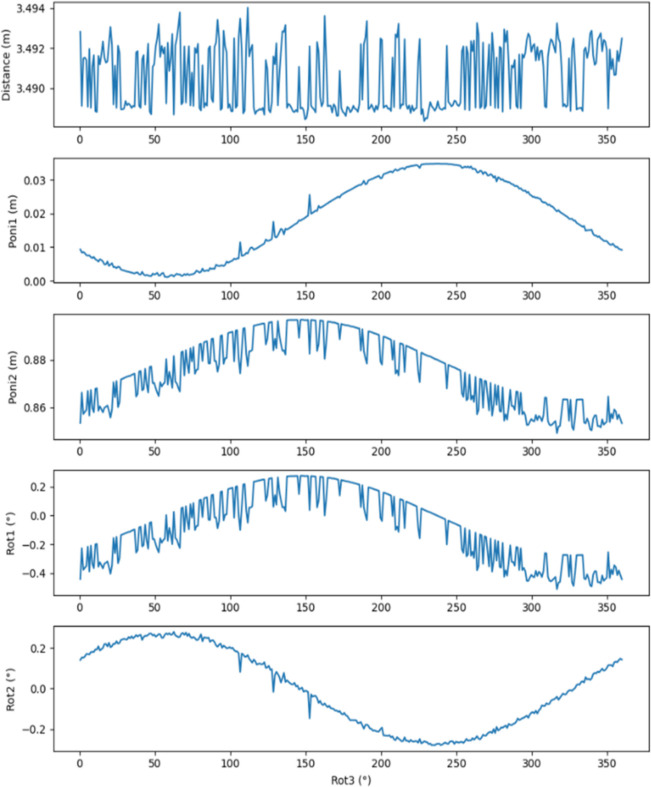
Rerunning calibration with addition of the precessing ( 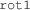 , 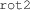 ) rotation centre parameter and with rotation-affected 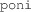 and 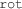 parameters. Note that the output does still show a cyclic behaviour, but not for distance, in contrast to the Fig. 5 ▸ output, but rather between 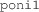 and 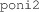 (as the beam centre precess about the rotation centre), with distance essentially constant, as anticipated. Notice also that both pairs – 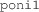 and 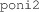 , and 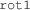 and 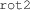 – differ in phase by 90°.

**Figure 7:**
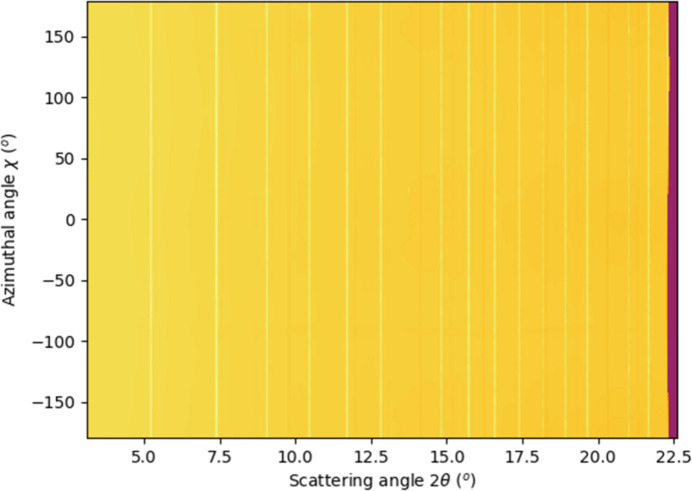
Two-dimensional azimuthally resolved polar integration of corrected diffraction data, at *E* = 52.7 keV and detx = 3500 mm.

**Figure 8:**
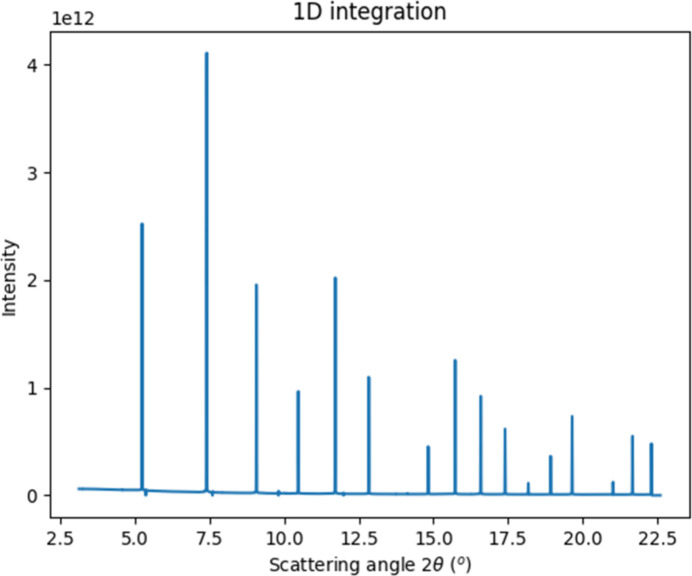
One-dimensional integration of the full solid-angle dataset shown in Fig. 7 ▸.

**Figure 9:**
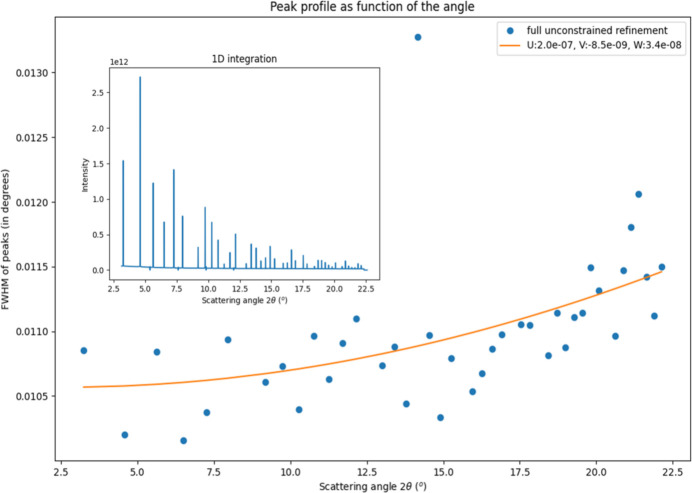
High-resolution, to 0.65 Å, data collected at highest energy and shorter detector distance (inset, at distance = 2150 mm), with integrated FWHM per peak of SRM660a with overlain estimated FWHM, from fitted *u*,*v*,*w* parameters. The outlying datum lies on a chip gap.
