# Supplementary material for: Correction and integration of solid-angle data from the azimuthally resolving 2D detector at ID06-LVP, ESRF
Source: J Synchrotron Radiat. 2023 Oct 17;30(Pt 6):1149–55. doi: 10.1107/S1600577523008020 (PMC10624032; doi:10.1107/S1600577523008020)
Supplement: Supplementary file 1 [file s-30-01149-sup1.pdf]

JOURNAL OF SYNCHROTRON RADIATION

Volume 31 (2024)

Supporting information for article:

Correction and integration of solid-angle data from the azimuthally resolving 2D detector at ID06-LVP, ESRF

Wilson A. Crichton, Jérôme Kieffer, Pierre Wattecamps, Valentin Valls, Gilles Berruyer, Marie Ruat and Vincent Favre-Nicolin

S1. Example script for correction, with integration and output of azimuthally resolving 2D and 1D datasets from the 900k-W detector output

Necessary raw data and script can be obtained from `https://zenodo.org/record/7957395`. See also the azimuthal correction file `yi5142sup2.html' in the online supporting information for this paper.

S2. Changes required for backwards compatibility of Detection Technology datasets, when one single image (of 512 lines) is output per full rotation

```
filename = "Y:/inhouse/2015/run1/lab6/512x1536.tif"       #full data
dark="Y:/inhouse/2015/run1/dk/dk_1536x512.tif"                 #dark
frames = fabio.open(filename).data
dk = fabio.open(dark).data
frames=frames-dk                                          #subtracted

rows_dir = 'C:/temp/rows'                          #junk directory for W
if not os.path.exists(rows_dir):
    os.makedirs(rows_dir)

for i, frame in enumerate(frames):                       # frame indexing
    img = numpy.broadcast_to(frame, (2, frame.shape[-1])) #2X height
    edf = fabio.edfimage.EdfImage(data=img)
    edf.save(f'{rows_dir}/[filename base]{i:03d}.edf')        #output
```

where the [*filename base*] matches the glob request in subsequent cells.
