# Supplementary material for: Correction and integration of solid-angle data from the azimuthally resolving 2D detector at ID06-LVP, ESRF
Source: J Synchrotron Radiat. 2023 Oct 17;30(Pt 6):1149–55. doi: 10.1107/S1600577523008020 (PMC10624032; doi:10.1107/S1600577523008020)
Supplement: Supplementary file 2 [file s-30-01149-sup2.html]

azimuthal\_correction


In [1]:

```
%matplotlib widget
```

In [2]:

```
import glob, copy
import numpy
import fabio
import pyFAI
import ipywidgets as widgets
from pyFAI.gui import jupyter
from matplotlib.pyplot import subplots
from matplotlib import pyplot as plt
from pyFAI.goniometer import SingleGeometry
from pyFAI.calibrant import get_calibrant
pi=numpy.pi
```

Frames for calibration with wildcard, will output total number of corresponding files and list of first 10

In [3]:

```
frames = glob.glob("SRM660a_31p8kev_3500mm_p900kw_0001_*.edf")
frames.sort()
print(len(frames))
frames[:10]
```

```
360
```

Out[3]:

```
['SRM660a_31p8keV_3500mm_p900kw_0001_0000_0000.edf',
 'SRM660a_31p8keV_3500mm_p900kw_0001_0000_0001.edf',
 'SRM660a_31p8keV_3500mm_p900kw_0001_0000_0002.edf',
 'SRM660a_31p8keV_3500mm_p900kw_0001_0000_0003.edf',
 'SRM660a_31p8keV_3500mm_p900kw_0001_0000_0004.edf',
 'SRM660a_31p8keV_3500mm_p900kw_0001_0000_0005.edf',
 'SRM660a_31p8keV_3500mm_p900kw_0001_0000_0006.edf',
 'SRM660a_31p8keV_3500mm_p900kw_0001_0000_0007.edf',
 'SRM660a_31p8keV_3500mm_p900kw_0001_0000_0008.edf',
 'SRM660a_31p8keV_3500mm_p900kw_0001_0000_0009.edf']
```

Shows the first frame

In [4]:

```
jupyter.display(fabio.open(frames[0]).data)
```

Out[4]:

```
<AxesSubplot:>
```

Figure

filename of initial pyfai.calib2 poni

In [5]:

```
ai = pyFAI.load("270.poni")
ai
```

Out[5]:

```
Detector Pilatus CdTe 900kw	 PixelSize= 1.720e-04, 1.720e-04 m
Wavelength= 3.898874e-11 m
SampleDetDist= 3.500529e+00 m	PONI= 8.131038e-02, 8.287482e-01 m	rot1=-0.007433  rot2=-0.017326  rot3=4.712389 rad
DirectBeamDist= 3501.152 mm	Center: x=4969.588, y=120.074 pix	Tilt= 1.080° tiltPlanRotation= -66.782° 𝛌= 0.390Å
```

add energy, if required

In [6]:

```
ai.energy = 31.8   #this needs pyfai 23.01
pilatus = ai.detector
```

Add calibration standard, outputs detail

In [7]:

```
LaB6 = get_calibrant("LaB6_SRM660a")
LaB6.set_wavelength = ai.wavelength
LaB6
```

Out[7]:

```
LaB6_SRM660a Calibrant with 91 reflections
```

Definition of pos, here in radians, from file number
Definition of recalibration of single frame geometry

In [8]:

```
def get_pos(frame):
    return numpy.deg2rad(float(frame.split(".")[-2].split("_")[-1]) + 0.5)
    
def recalib(frame):
    index = int(frame.split(".")[-2].split("_")[-1])
    img = fabio.open(frame).data #no metadata in the header
    sg = SingleGeometry(frame, img, frame, get_pos,None, LaB6, copy.copy(ai.detector), copy.copy(ai))
    sg.extract_cp(Imin=img.mean())
    sg.geometry_refinement.rot3 = get_pos(frame)
    sg.geometry_refinement.rot1 = 0
    sg.geometry_refinement.rot2 = 0
    sg.geometry_refinement.refine3(fix=["rot1", "rot2", "rot3", "wavelength"])
    return sg
```

First estimation of single frame geometries, with orthogonal detector

In [9]:

```
%time geometries = {f: recalib(f) for f in frames}
```

```
Wall time: 3min 56s
```

In [10]:

```
ax = jupyter.display(sg=geometries[frames[270]])
ax.legend().set_visible(False)
```

Figure

Output of first refinement, orthogonal position

In [11]:

```
fig,ax = subplots(3, figsize=(9,9))
angles = [numpy.rad2deg(get_pos(frame)) for frame in frames]
dist = numpy.array([geometries[frame].geometry_refinement.dist for frame in frames])
poni1 = numpy.array([geometries[frame].geometry_refinement.poni1 for frame in frames])
poni2 = numpy.array([geometries[frame].geometry_refinement.poni2 for frame in frames])
ax[0].plot(angles, dist)
ax[1].plot(angles, poni1)
ax[2].plot(angles, poni2)
ax[0].set_ylabel("Distance (m)")
ax[1].set_ylabel("Poni1 (m)")
ax[2].set_ylabel("Poni2 (m)")
ax[2].set_xlabel("Azimuth, °")
plt.tight_layout()
```

Figure

Check control points and estimation of geometry per frame with slider

In [12]:

```
fig, ax = subplots(figsize=(10,3))
ax = jupyter.display(sg=geometries[frames[0]], ax=ax)
ax.set_title(frames[0])
ax.legend().set_visible(False)

def update(change):
    idx = change["new"]
    frame=frames[idx]
    sg = geometries[frame]
    img = sg.image
    ax.images[0].set_data(img)
    ax.set_title(frame)
    ax.collections.clear()
    cp = sg.control_points
    for lbl in cp.get_labels():
            pt = numpy.array(cp.get(lbl=lbl).points)
            if len(pt) > 0:
                ax.scatter(pt[:, 1], pt[:, 0], label=lbl)
    fig.canvas.draw()

int_slider = widgets.IntSlider(value=0, min=0, max=len(frames)-1, step=1,    
                               description='Angle', continuous_update=False)
int_slider.observe(update, names="value")
display(int_slider)
```

```
IntSlider(value=0, continuous_update=False, description='Angle', max=359)
```

Figure

Start of goniometer definition

In [13]:

```
from pyFAI.goniometer import GoniometerRefinement, GeometryTransformation
```

Addition of rotation centre as point and rotations about poni1, poni2, rot1 and rot2 as expressions, definition of rot3 as pos, set initial from singlegeometry refinement and add bounds to fit params

In [14]:

```
goniotrans = GeometryTransformation(param_names = ["dist", 
                                                   "poni1",
                                                   "poni2",
                                                   "rot1", 
                                                   "rot2", 
                                                   "rot_x", "rot_y"], #rotation centre 
                                    dist_expr= "dist", 
                                    poni1_expr= "poni1 + ((rot_x*cos(2*pi-(pos))) - rot_y*sin(2*pi-(pos)))",
                                    poni2_expr= "poni2 + ((rot_x*sin(2*pi-(pos))) + rot_y*cos(2*pi-(pos)))",
                                    rot1_expr= "(rot1*cos(2*pi-(pos)) - rot2*sin(2*pi-(pos)))", 
                                    rot2_expr= "(rot1*sin(2*pi-(pos)) + rot2*cos(2*pi-(pos)))",
                                    rot3_expr= "pos")                           
#initial positions, from sg 
param = {"dist":numpy.median(dist),
         "poni1": numpy.median(poni1),
         "poni2":numpy.median(poni2),
         "rot1": 0,
         "rot2": 0,
         "rot_x": 0,
         "rot_y": 0
        }
#limits rangs
bounds = {"dist": (1, 5),
          "poni1": (0, 2),
          "poni2": (0, 2),
          "rot1": (-1,1),
          "rot2": (-1,1),
          "rot_x":(-0.1,0.1), #metres
          "rot_y":(-0.1,0.1)
         }
gonioref = GoniometerRefinement(param, #initial guess
                                bounds=bounds,
                                pos_function=get_pos,
                                trans_function=goniotrans,
                                detector=ai.detector, wavelength=ai.wavelength)
#param, bounds
```

In [15]:

```
print("Empty refinement object:", gonioref)

#Let's populate the goniometer refinement object with all control point files:
gonioref.single_geometries.update(geometries)
    

print("Filled refinement object:")
print(gonioref)
```

```
Empty refinement object: GoniometerRefinement with 0 geometries labeled: .
Filled refinement object:
GoniometerRefinement with 360 geometries labeled: SRM660a_31p8keV_3500mm_p900kw_0001_0000_0000.edf, SRM660a_31p8keV_3500mm_p900kw_0001_0000_0001.edf, SRM660a_31p8keV_3500mm_p900kw_0001_0000_0002.edf, SRM660a_31p8keV_3500mm_p900kw_0001_0000_0003.edf, SRM660a_31p8keV_3500mm_p900kw_0001_0000_0004.edf, SRM660a_31p8keV_3500mm_p900kw_0001_0000_0005.edf, SRM660a_31p8keV_3500mm_p900kw_0001_0000_0006.edf, SRM660a_31p8keV_3500mm_p900kw_0001_0000_0007.edf, SRM660a_31p8keV_3500mm_p900kw_0001_0000_0008.edf, SRM660a_31p8keV_3500mm_p900kw_0001_0000_0009.edf, SRM660a_31p8keV_3500mm_p900kw_0001_0000_0010.edf, SRM660a_31p8keV_3500mm_p900kw_0001_0000_0011.edf, SRM660a_31p8keV_3500mm_p900kw_0001_0000_0012.edf, SRM660a_31p8keV_3500mm_p900kw_0001_0000_0013.edf, SRM660a_31p8keV_3500mm_p900kw_0001_0000_0014.edf, SRM660a_31p8keV_3500mm_p900kw_0001_0000_0015.edf, SRM660a_31p8keV_3500mm_p900kw_0001_0000_0016.edf, SRM660a_31p8keV_3500mm_p900kw_0001_0000_0017.edf, SRM660a_31p8keV_3500mm_p900kw_0001_0000_0018.edf, SRM660a_31p8keV_3500mm_p900kw_0001_0000_0019.edf, SRM660a_31p8keV_3500mm_p900kw_0001_0000_0020.edf, SRM660a_31p8keV_3500mm_p900kw_0001_0000_0021.edf, SRM660a_31p8keV_3500mm_p900kw_0001_0000_0022.edf, SRM660a_31p8keV_3500mm_p900kw_0001_0000_0023.edf, SRM660a_31p8keV_3500mm_p900kw_0001_0000_0024.edf, SRM660a_31p8keV_3500mm_p900kw_0001_0000_0025.edf, SRM660a_31p8keV_3500mm_p900kw_0001_0000_0026.edf, SRM660a_31p8keV_3500mm_p900kw_0001_0000_0027.edf, SRM660a_31p8keV_3500mm_p900kw_0001_0000_0028.edf, SRM660a_31p8keV_3500mm_p900kw_0001_0000_0029.edf, SRM660a_31p8keV_3500mm_p900kw_0001_0000_0030.edf, SRM660a_31p8keV_3500mm_p900kw_0001_0000_0031.edf, SRM660a_31p8keV_3500mm_p900kw_0001_0000_0032.edf, SRM660a_31p8keV_3500mm_p900kw_0001_0000_0033.edf, SRM660a_31p8keV_3500mm_p900kw_0001_0000_0034.edf, SRM660a_31p8keV_3500mm_p900kw_0001_0000_0035.edf, SRM660a_31p8keV_3500mm_p900kw_0001_0000_0036.edf, SRM660a_31p8keV_3500mm_p900kw_0001_0000_0037.edf, SRM660a_31p8keV_3500mm_p900kw_0001_0000_0038.edf, SRM660a_31p8keV_3500mm_p900kw_0001_0000_0039.edf, SRM660a_31p8keV_3500mm_p900kw_0001_0000_0040.edf, SRM660a_31p8keV_3500mm_p900kw_0001_0000_0041.edf, SRM660a_31p8keV_3500mm_p900kw_0001_0000_0042.edf, SRM660a_31p8keV_3500mm_p900kw_0001_0000_0043.edf, SRM660a_31p8keV_3500mm_p900kw_0001_0000_0044.edf, SRM660a_31p8keV_3500mm_p900kw_0001_0000_0045.edf, SRM660a_31p8keV_3500mm_p900kw_0001_0000_0046.edf, SRM660a_31p8keV_3500mm_p900kw_0001_0000_0047.edf, SRM660a_31p8keV_3500mm_p900kw_0001_0000_0048.edf, SRM660a_31p8keV_3500mm_p900kw_0001_0000_0049.edf, SRM660a_31p8keV_3500mm_p900kw_0001_0000_0050.edf, SRM660a_31p8keV_3500mm_p900kw_0001_0000_0051.edf, SRM660a_31p8keV_3500mm_p900kw_0001_0000_0052.edf, SRM660a_31p8keV_3500mm_p900kw_0001_0000_0053.edf, SRM660a_31p8keV_3500mm_p900kw_0001_0000_0054.edf, SRM660a_31p8keV_3500mm_p900kw_0001_0000_0055.edf, SRM660a_31p8keV_3500mm_p900kw_0001_0000_0056.edf, SRM660a_31p8keV_3500mm_p900kw_0001_0000_0057.edf, SRM660a_31p8keV_3500mm_p900kw_0001_0000_0058.edf, SRM660a_31p8keV_3500mm_p900kw_0001_0000_0059.edf, SRM660a_31p8keV_3500mm_p900kw_0001_0000_0060.edf, SRM660a_31p8keV_3500mm_p900kw_0001_0000_0061.edf, SRM660a_31p8keV_3500mm_p900kw_0001_0000_0062.edf, SRM660a_31p8keV_3500mm_p900kw_0001_0000_0063.edf, SRM660a_31p8keV_3500mm_p900kw_0001_0000_0064.edf, SRM660a_31p8keV_3500mm_p900kw_0001_0000_0065.edf, SRM660a_31p8keV_3500mm_p900kw_0001_0000_0066.edf, SRM660a_31p8keV_3500mm_p900kw_0001_0000_0067.edf, SRM660a_31p8keV_3500mm_p900kw_0001_0000_0068.edf, SRM660a_31p8keV_3500mm_p900kw_0001_0000_0069.edf, SRM660a_31p8keV_3500mm_p900kw_0001_0000_0070.edf, SRM660a_31p8keV_3500mm_p900kw_0001_0000_0071.edf, SRM660a_31p8keV_3500mm_p900kw_0001_0000_0072.edf, SRM660a_31p8keV_3500mm_p900kw_0001_0000_0073.edf, SRM660a_31p8keV_3500mm_p900kw_0001_0000_0074.edf, SRM660a_31p8keV_3500mm_p900kw_0001_0000_0075.edf, SRM660a_31p8keV_3500mm_p900kw_0001_0000_0076.edf, SRM660a_31p8keV_3500mm_p900kw_0001_0000_0077.edf, SRM660a_31p8keV_3500mm_p900kw_0001_0000_0078.edf, SRM660a_31p8keV_3500mm_p900kw_0001_0000_0079.edf, SRM660a_31p8keV_3500mm_p900kw_0001_0000_0080.edf, SRM660a_31p8keV_3500mm_p900kw_0001_0000_0081.edf, SRM660a_31p8keV_3500mm_p900kw_0001_0000_0082.edf, SRM660a_31p8keV_3500mm_p900kw_0001_0000_0083.edf, SRM660a_31p8keV_3500mm_p900kw_0001_0000_0084.edf, SRM660a_31p8keV_3500mm_p900kw_0001_0000_0085.edf, SRM660a_31p8keV_3500mm_p900kw_0001_0000_0086.edf, SRM660a_31p8keV_3500mm_p900kw_0001_0000_0087.edf, SRM660a_31p8keV_3500mm_p900kw_0001_0000_0088.edf, SRM660a_31p8keV_3500mm_p900kw_0001_0000_0089.edf, SRM660a_31p8keV_3500mm_p900kw_0001_0000_0090.edf, SRM660a_31p8keV_3500mm_p900kw_0001_0000_0091.edf, SRM660a_31p8keV_3500mm_p900kw_0001_0000_0092.edf, SRM660a_31p8keV_3500mm_p900kw_0001_0000_0093.edf, SRM660a_31p8keV_3500mm_p900kw_0001_0000_0094.edf, SRM660a_31p8keV_3500mm_p900kw_0001_0000_0095.edf, SRM660a_31p8keV_3500mm_p900kw_0001_0000_0096.edf, SRM660a_31p8keV_3500mm_p900kw_0001_0000_0097.edf, SRM660a_31p8keV_3500mm_p900kw_0001_0000_0098.edf, SRM660a_31p8keV_3500mm_p900kw_0001_0000_0099.edf, SRM660a_31p8keV_3500mm_p900kw_0001_0000_0100.edf, SRM660a_31p8keV_3500mm_p900kw_0001_0000_0101.edf, SRM660a_31p8keV_3500mm_p900kw_0001_0000_0102.edf, SRM660a_31p8keV_3500mm_p900kw_0001_0000_0103.edf, SRM660a_31p8keV_3500mm_p900kw_0001_0000_0104.edf, SRM660a_31p8keV_3500mm_p900kw_0001_0000_0105.edf, SRM660a_31p8keV_3500mm_p900kw_0001_0000_0106.edf, SRM660a_31p8keV_3500mm_p900kw_0001_0000_0107.edf, SRM660a_31p8keV_3500mm_p900kw_0001_0000_0108.edf, SRM660a_31p8keV_3500mm_p900kw_0001_0000_0109.edf, SRM660a_31p8keV_3500mm_p900kw_0001_0000_0110.edf, SRM660a_31p8keV_3500mm_p900kw_0001_0000_0111.edf, SRM660a_31p8keV_3500mm_p900kw_0001_0000_0112.edf, SRM660a_31p8keV_3500mm_p900kw_0001_0000_0113.edf, SRM660a_31p8keV_3500mm_p900kw_0001_0000_0114.edf, SRM660a_31p8keV_3500mm_p900kw_0001_0000_0115.edf, SRM660a_31p8keV_3500mm_p900kw_0001_0000_0116.edf, SRM660a_31p8keV_3500mm_p900kw_0001_0000_0117.edf, SRM660a_31p8keV_3500mm_p900kw_0001_0000_0118.edf, SRM660a_31p8keV_3500mm_p900kw_0001_0000_0119.edf, SRM660a_31p8keV_3500mm_p900kw_0001_0000_0120.edf, SRM660a_31p8keV_3500mm_p900kw_0001_0000_0121.edf, SRM660a_31p8keV_3500mm_p900kw_0001_0000_0122.edf, SRM660a_31p8keV_3500mm_p900kw_0001_0000_0123.edf, SRM660a_31p8keV_3500mm_p900kw_0001_0000_0124.edf, SRM660a_31p8keV_3500mm_p900kw_0001_0000_0125.edf, SRM660a_31p8keV_3500mm_p900kw_0001_0000_0126.edf, SRM660a_31p8keV_3500mm_p900kw_0001_0000_0127.edf, SRM660a_31p8keV_3500mm_p900kw_0001_0000_0128.edf, SRM660a_31p8keV_3500mm_p900kw_0001_0000_0129.edf, SRM660a_31p8keV_3500mm_p900kw_0001_0000_0130.edf, SRM660a_31p8keV_3500mm_p900kw_0001_0000_0131.edf, SRM660a_31p8keV_3500mm_p900kw_0001_0000_0132.edf, SRM660a_31p8keV_3500mm_p900kw_0001_0000_0133.edf, SRM660a_31p8keV_3500mm_p900kw_0001_0000_0134.edf, SRM660a_31p8keV_3500mm_p900kw_0001_0000_0135.edf, SRM660a_31p8keV_3500mm_p900kw_0001_0000_0136.edf, SRM660a_31p8keV_3500mm_p900kw_0001_0000_0137.edf, SRM660a_31p8keV_3500mm_p900kw_0001_0000_0138.edf, SRM660a_31p8keV_3500mm_p900kw_0001_0000_0139.edf, SRM660a_31p8keV_3500mm_p900kw_0001_0000_0140.edf, SRM660a_31p8keV_3500mm_p900kw_0001_0000_0141.edf, SRM660a_31p8keV_3500mm_p900kw_0001_0000_0142.edf, SRM660a_31p8keV_3500mm_p900kw_0001_0000_0143.edf, SRM660a_31p8keV_3500mm_p900kw_0001_0000_0144.edf, SRM660a_31p8keV_3500mm_p900kw_0001_0000_0145.edf, SRM660a_31p8keV_3500mm_p900kw_0001_0000_0146.edf, SRM660a_31p8keV_3500mm_p900kw_0001_0000_0147.edf, SRM660a_31p8keV_3500mm_p900kw_0001_0000_0148.edf, SRM660a_31p8keV_3500mm_p900kw_0001_0000_0149.edf, SRM660a_31p8keV_3500mm_p900kw_0001_0000_0150.edf, SRM660a_31p8keV_3500mm_p900kw_0001_0000_0151.edf, SRM660a_31p8keV_3500mm_p900kw_0001_0000_0152.edf, SRM660a_31p8keV_3500mm_p900kw_0001_0000_0153.edf, SRM660a_31p8keV_3500mm_p900kw_0001_0000_0154.edf, SRM660a_31p8keV_3500mm_p900kw_0001_0000_0155.edf, SRM660a_31p8keV_3500mm_p900kw_0001_0000_0156.edf, SRM660a_31p8keV_3500mm_p900kw_0001_0000_0157.edf, SRM660a_31p8keV_3500mm_p900kw_0001_0000_0158.edf, SRM660a_31p8keV_3500mm_p900kw_0001_0000_0159.edf, SRM660a_31p8keV_3500mm_p900kw_0001_0000_0160.edf, SRM660a_31p8keV_3500mm_p900kw_0001_0000_0161.edf, SRM660a_31p8keV_3500mm_p900kw_0001_0000_0162.edf, SRM660a_31p8keV_3500mm_p900kw_0001_0000_0163.edf, SRM660a_31p8keV_3500mm_p900kw_0001_0000_0164.edf, SRM660a_31p8keV_3500mm_p900kw_0001_0000_0165.edf, SRM660a_31p8keV_3500mm_p900kw_0001_0000_0166.edf, SRM660a_31p8keV_3500mm_p900kw_0001_0000_0167.edf, SRM660a_31p8keV_3500mm_p900kw_0001_0000_0168.edf, SRM660a_31p8keV_3500mm_p900kw_0001_0000_0169.edf, SRM660a_31p8keV_3500mm_p900kw_0001_0000_0170.edf, SRM660a_31p8keV_3500mm_p900kw_0001_0000_0171.edf, SRM660a_31p8keV_3500mm_p900kw_0001_0000_0172.edf, SRM660a_31p8keV_3500mm_p900kw_0001_0000_0173.edf, SRM660a_31p8keV_3500mm_p900kw_0001_0000_0174.edf, SRM660a_31p8keV_3500mm_p900kw_0001_0000_0175.edf, SRM660a_31p8keV_3500mm_p900kw_0001_0000_0176.edf, SRM660a_31p8keV_3500mm_p900kw_0001_0000_0177.edf, SRM660a_31p8keV_3500mm_p900kw_0001_0000_0178.edf, SRM660a_31p8keV_3500mm_p900kw_0001_0000_0179.edf, SRM660a_31p8keV_3500mm_p900kw_0001_0000_0180.edf, SRM660a_31p8keV_3500mm_p900kw_0001_0000_0181.edf, SRM660a_31p8keV_3500mm_p900kw_0001_0000_0182.edf, SRM660a_31p8keV_3500mm_p900kw_0001_0000_0183.edf, SRM660a_31p8keV_3500mm_p900kw_0001_0000_0184.edf, SRM660a_31p8keV_3500mm_p900kw_0001_0000_0185.edf, SRM660a_31p8keV_3500mm_p900kw_0001_0000_0186.edf, SRM660a_31p8keV_3500mm_p900kw_0001_0000_0187.edf, SRM660a_31p8keV_3500mm_p900kw_0001_0000_0188.edf, SRM660a_31p8keV_3500mm_p900kw_0001_0000_0189.edf, SRM660a_31p8keV_3500mm_p900kw_0001_0000_0190.edf, SRM660a_31p8keV_3500mm_p900kw_0001_0000_0191.edf, SRM660a_31p8keV_3500mm_p900kw_0001_0000_0192.edf, SRM660a_31p8keV_3500mm_p900kw_0001_0000_0193.edf, SRM660a_31p8keV_3500mm_p900kw_0001_0000_0194.edf, SRM660a_31p8keV_3500mm_p900kw_0001_0000_0195.edf, SRM660a_31p8keV_3500mm_p900kw_0001_0000_0196.edf, SRM660a_31p8keV_3500mm_p900kw_0001_0000_0197.edf, SRM660a_31p8keV_3500mm_p900kw_0001_0000_0198.edf, SRM660a_31p8keV_3500mm_p900kw_0001_0000_0199.edf, SRM660a_31p8keV_3500mm_p900kw_0001_0000_0200.edf, SRM660a_31p8keV_3500mm_p900kw_0001_0000_0201.edf, SRM660a_31p8keV_3500mm_p900kw_0001_0000_0202.edf, SRM660a_31p8keV_3500mm_p900kw_0001_0000_0203.edf, SRM660a_31p8keV_3500mm_p900kw_0001_0000_0204.edf, SRM660a_31p8keV_3500mm_p900kw_0001_0000_0205.edf, SRM660a_31p8keV_3500mm_p900kw_0001_0000_0206.edf, SRM660a_31p8keV_3500mm_p900kw_0001_0000_0207.edf, SRM660a_31p8keV_3500mm_p900kw_0001_0000_0208.edf, SRM660a_31p8keV_3500mm_p900kw_0001_0000_0209.edf, SRM660a_31p8keV_3500mm_p900kw_0001_0000_0210.edf, SRM660a_31p8keV_3500mm_p900kw_0001_0000_0211.edf, SRM660a_31p8keV_3500mm_p900kw_0001_0000_0212.edf, SRM660a_31p8keV_3500mm_p900kw_0001_0000_0213.edf, SRM660a_31p8keV_3500mm_p900kw_0001_0000_0214.edf, SRM660a_31p8keV_3500mm_p900kw_0001_0000_0215.edf, SRM660a_31p8keV_3500mm_p900kw_0001_0000_0216.edf, SRM660a_31p8keV_3500mm_p900kw_0001_0000_0217.edf, SRM660a_31p8keV_3500mm_p900kw_0001_0000_0218.edf, SRM660a_31p8keV_3500mm_p900kw_0001_0000_0219.edf, SRM660a_31p8keV_3500mm_p900kw_0001_0000_0220.edf, SRM660a_31p8keV_3500mm_p900kw_0001_0000_0221.edf, SRM660a_31p8keV_3500mm_p900kw_0001_0000_0222.edf, SRM660a_31p8keV_3500mm_p900kw_0001_0000_0223.edf, SRM660a_31p8keV_3500mm_p900kw_0001_0000_0224.edf, SRM660a_31p8keV_3500mm_p900kw_0001_0000_0225.edf, SRM660a_31p8keV_3500mm_p900kw_0001_0000_0226.edf, SRM660a_31p8keV_3500mm_p900kw_0001_0000_0227.edf, SRM660a_31p8keV_3500mm_p900kw_0001_0000_0228.edf, SRM660a_31p8keV_3500mm_p900kw_0001_0000_0229.edf, SRM660a_31p8keV_3500mm_p900kw_0001_0000_0230.edf, SRM660a_31p8keV_3500mm_p900kw_0001_0000_0231.edf, SRM660a_31p8keV_3500mm_p900kw_0001_0000_0232.edf, SRM660a_31p8keV_3500mm_p900kw_0001_0000_0233.edf, SRM660a_31p8keV_3500mm_p900kw_0001_0000_0234.edf, SRM660a_31p8keV_3500mm_p900kw_0001_0000_0235.edf, SRM660a_31p8keV_3500mm_p900kw_0001_0000_0236.edf, SRM660a_31p8keV_3500mm_p900kw_0001_0000_0237.edf, SRM660a_31p8keV_3500mm_p900kw_0001_0000_0238.edf, SRM660a_31p8keV_3500mm_p900kw_0001_0000_0239.edf, SRM660a_31p8keV_3500mm_p900kw_0001_0000_0240.edf, SRM660a_31p8keV_3500mm_p900kw_0001_0000_0241.edf, SRM660a_31p8keV_3500mm_p900kw_0001_0000_0242.edf, SRM660a_31p8keV_3500mm_p900kw_0001_0000_0243.edf, SRM660a_31p8keV_3500mm_p900kw_0001_0000_0244.edf, SRM660a_31p8keV_3500mm_p900kw_0001_0000_0245.edf, SRM660a_31p8keV_3500mm_p900kw_0001_0000_0246.edf, SRM660a_31p8keV_3500mm_p900kw_0001_0000_0247.edf, SRM660a_31p8keV_3500mm_p900kw_0001_0000_0248.edf, SRM660a_31p8keV_3500mm_p900kw_0001_0000_0249.edf, SRM660a_31p8keV_3500mm_p900kw_0001_0000_0250.edf, SRM660a_31p8keV_3500mm_p900kw_0001_0000_0251.edf, SRM660a_31p8keV_3500mm_p900kw_0001_0000_0252.edf, SRM660a_31p8keV_3500mm_p900kw_0001_0000_0253.edf, SRM660a_31p8keV_3500mm_p900kw_0001_0000_0254.edf, SRM660a_31p8keV_3500mm_p900kw_0001_0000_0255.edf, SRM660a_31p8keV_3500mm_p900kw_0001_0000_0256.edf, SRM660a_31p8keV_3500mm_p900kw_0001_0000_0257.edf, SRM660a_31p8keV_3500mm_p900kw_0001_0000_0258.edf, SRM660a_31p8keV_3500mm_p900kw_0001_0000_0259.edf, SRM660a_31p8keV_3500mm_p900kw_0001_0000_0260.edf, SRM660a_31p8keV_3500mm_p900kw_0001_0000_0261.edf, SRM660a_31p8keV_3500mm_p900kw_0001_0000_0262.edf, SRM660a_31p8keV_3500mm_p900kw_0001_0000_0263.edf, SRM660a_31p8keV_3500mm_p900kw_0001_0000_0264.edf, SRM660a_31p8keV_3500mm_p900kw_0001_0000_0265.edf, SRM660a_31p8keV_3500mm_p900kw_0001_0000_0266.edf, SRM660a_31p8keV_3500mm_p900kw_0001_0000_0267.edf, SRM660a_31p8keV_3500mm_p900kw_0001_0000_0268.edf, SRM660a_31p8keV_3500mm_p900kw_0001_0000_0269.edf, SRM660a_31p8keV_3500mm_p900kw_0001_0000_0270.edf, SRM660a_31p8keV_3500mm_p900kw_0001_0000_0271.edf, SRM660a_31p8keV_3500mm_p900kw_0001_0000_0272.edf, SRM660a_31p8keV_3500mm_p900kw_0001_0000_0273.edf, SRM660a_31p8keV_3500mm_p900kw_0001_0000_0274.edf, SRM660a_31p8keV_3500mm_p900kw_0001_0000_0275.edf, SRM660a_31p8keV_3500mm_p900kw_0001_0000_0276.edf, SRM660a_31p8keV_3500mm_p900kw_0001_0000_0277.edf, SRM660a_31p8keV_3500mm_p900kw_0001_0000_0278.edf, SRM660a_31p8keV_3500mm_p900kw_0001_0000_0279.edf, SRM660a_31p8keV_3500mm_p900kw_0001_0000_0280.edf, SRM660a_31p8keV_3500mm_p900kw_0001_0000_0281.edf, SRM660a_31p8keV_3500mm_p900kw_0001_0000_0282.edf, SRM660a_31p8keV_3500mm_p900kw_0001_0000_0283.edf, SRM660a_31p8keV_3500mm_p900kw_0001_0000_0284.edf, SRM660a_31p8keV_3500mm_p900kw_0001_0000_0285.edf, SRM660a_31p8keV_3500mm_p900kw_0001_0000_0286.edf, SRM660a_31p8keV_3500mm_p900kw_0001_0000_0287.edf, SRM660a_31p8keV_3500mm_p900kw_0001_0000_0288.edf, SRM660a_31p8keV_3500mm_p900kw_0001_0000_0289.edf, SRM660a_31p8keV_3500mm_p900kw_0001_0000_0290.edf, SRM660a_31p8keV_3500mm_p900kw_0001_0000_0291.edf, SRM660a_31p8keV_3500mm_p900kw_0001_0000_0292.edf, SRM660a_31p8keV_3500mm_p900kw_0001_0000_0293.edf, SRM660a_31p8keV_3500mm_p900kw_0001_0000_0294.edf, SRM660a_31p8keV_3500mm_p900kw_0001_0000_0295.edf, SRM660a_31p8keV_3500mm_p900kw_0001_0000_0296.edf, SRM660a_31p8keV_3500mm_p900kw_0001_0000_0297.edf, SRM660a_31p8keV_3500mm_p900kw_0001_0000_0298.edf, SRM660a_31p8keV_3500mm_p900kw_0001_0000_0299.edf, SRM660a_31p8keV_3500mm_p900kw_0001_0000_0300.edf, SRM660a_31p8keV_3500mm_p900kw_0001_0000_0301.edf, SRM660a_31p8keV_3500mm_p900kw_0001_0000_0302.edf, SRM660a_31p8keV_3500mm_p900kw_0001_0000_0303.edf, SRM660a_31p8keV_3500mm_p900kw_0001_0000_0304.edf, SRM660a_31p8keV_3500mm_p900kw_0001_0000_0305.edf, SRM660a_31p8keV_3500mm_p900kw_0001_0000_0306.edf, SRM660a_31p8keV_3500mm_p900kw_0001_0000_0307.edf, SRM660a_31p8keV_3500mm_p900kw_0001_0000_0308.edf, SRM660a_31p8keV_3500mm_p900kw_0001_0000_0309.edf, SRM660a_31p8keV_3500mm_p900kw_0001_0000_0310.edf, SRM660a_31p8keV_3500mm_p900kw_0001_0000_0311.edf, SRM660a_31p8keV_3500mm_p900kw_0001_0000_0312.edf, SRM660a_31p8keV_3500mm_p900kw_0001_0000_0313.edf, SRM660a_31p8keV_3500mm_p900kw_0001_0000_0314.edf, SRM660a_31p8keV_3500mm_p900kw_0001_0000_0315.edf, SRM660a_31p8keV_3500mm_p900kw_0001_0000_0316.edf, SRM660a_31p8keV_3500mm_p900kw_0001_0000_0317.edf, SRM660a_31p8keV_3500mm_p900kw_0001_0000_0318.edf, SRM660a_31p8keV_3500mm_p900kw_0001_0000_0319.edf, SRM660a_31p8keV_3500mm_p900kw_0001_0000_0320.edf, SRM660a_31p8keV_3500mm_p900kw_0001_0000_0321.edf, SRM660a_31p8keV_3500mm_p900kw_0001_0000_0322.edf, SRM660a_31p8keV_3500mm_p900kw_0001_0000_0323.edf, SRM660a_31p8keV_3500mm_p900kw_0001_0000_0324.edf, SRM660a_31p8keV_3500mm_p900kw_0001_0000_0325.edf, SRM660a_31p8keV_3500mm_p900kw_0001_0000_0326.edf, SRM660a_31p8keV_3500mm_p900kw_0001_0000_0327.edf, SRM660a_31p8keV_3500mm_p900kw_0001_0000_0328.edf, SRM660a_31p8keV_3500mm_p900kw_0001_0000_0329.edf, SRM660a_31p8keV_3500mm_p900kw_0001_0000_0330.edf, SRM660a_31p8keV_3500mm_p900kw_0001_0000_0331.edf, SRM660a_31p8keV_3500mm_p900kw_0001_0000_0332.edf, SRM660a_31p8keV_3500mm_p900kw_0001_0000_0333.edf, SRM660a_31p8keV_3500mm_p900kw_0001_0000_0334.edf, SRM660a_31p8keV_3500mm_p900kw_0001_0000_0335.edf, SRM660a_31p8keV_3500mm_p900kw_0001_0000_0336.edf, SRM660a_31p8keV_3500mm_p900kw_0001_0000_0337.edf, SRM660a_31p8keV_3500mm_p900kw_0001_0000_0338.edf, SRM660a_31p8keV_3500mm_p900kw_0001_0000_0339.edf, SRM660a_31p8keV_3500mm_p900kw_0001_0000_0340.edf, SRM660a_31p8keV_3500mm_p900kw_0001_0000_0341.edf, SRM660a_31p8keV_3500mm_p900kw_0001_0000_0342.edf, SRM660a_31p8keV_3500mm_p900kw_0001_0000_0343.edf, SRM660a_31p8keV_3500mm_p900kw_0001_0000_0344.edf, SRM660a_31p8keV_3500mm_p900kw_0001_0000_0345.edf, SRM660a_31p8keV_3500mm_p900kw_0001_0000_0346.edf, SRM660a_31p8keV_3500mm_p900kw_0001_0000_0347.edf, SRM660a_31p8keV_3500mm_p900kw_0001_0000_0348.edf, SRM660a_31p8keV_3500mm_p900kw_0001_0000_0349.edf, SRM660a_31p8keV_3500mm_p900kw_0001_0000_0350.edf, SRM660a_31p8keV_3500mm_p900kw_0001_0000_0351.edf, SRM660a_31p8keV_3500mm_p900kw_0001_0000_0352.edf, SRM660a_31p8keV_3500mm_p900kw_0001_0000_0353.edf, SRM660a_31p8keV_3500mm_p900kw_0001_0000_0354.edf, SRM660a_31p8keV_3500mm_p900kw_0001_0000_0355.edf, SRM660a_31p8keV_3500mm_p900kw_0001_0000_0356.edf, SRM660a_31p8keV_3500mm_p900kw_0001_0000_0357.edf, SRM660a_31p8keV_3500mm_p900kw_0001_0000_0358.edf, SRM660a_31p8keV_3500mm_p900kw_0001_0000_0359.edf.
```

In [16]:

```
# Initial refinement of the goniometer model with 5 dof

gonioref.refine3()
```

```
Free parameters: ['dist', 'poni1', 'poni2', 'rot1', 'rot2', 'rot_x', 'rot_y']
Fixed: {}
     fun: 4.322561930712424e-09
     jac: array([-1.30505966e-07, -3.50683987e-09, -8.53609178e-09,  2.83069498e-07,
       -1.21447207e-07,  1.35054182e-07,  1.73424320e-07])
 message: 'Optimization terminated successfully'
    nfev: 40
     nit: 5
    njev: 5
  status: 0
 success: True
       x: array([ 3.49562373e+00,  1.82324565e-02,  8.54504224e-01, -7.73635898e-05,
        2.78533566e-05,  6.23964988e-06,  1.92785654e-05])
Constrained Least square 8.214511938359905e-09 --> 4.322561930712424e-09
maxdelta on rot1: 0.0 --> -7.736358978451682e-05
```

Out[16]:

```
4.322561930712424e-09
```

In [17]:

```
for k,v in zip(gonioref.nt_param._fields, gonioref.param):
    print(f"{k:>12s}: {v}")
```

```
        dist: 3.495623729290002
       poni1: 0.01823245645308157
       poni2: 0.8545042239063068
        rot1: -7.736358978451682e-05
        rot2: 2.785335659132407e-05
       rot_x: 6.239649882104696e-06
       rot_y: 1.9278565392570917e-05
```

In [18]:

```
nplot = 4
fig, ax = subplots(nplot, figsize=(12,1*nplot))
for i in range(nplot):
    idx = i*360//nplot
    frame = frames[idx]
    sg = gonioref.single_geometries[frame]
    sg.geometry_refinement.set_param(gonioref.get_ai(sg.get_position()).param)
    axi = jupyter.display(sg=sg, ax=ax[i])
    axi.set_title(frames[idx])
    axi.legend().set_visible(False)
plt.tight_layout()
```

Figure

In [19]:

```
%%time
fraction = 10
angles = []
dist = []
poni1 = []
poni2 = []
rot1 = []
rot2 = []
rot_x = []
rot_y = []
for idx in range(0,len(frames),fraction):
    frame = frames[idx]
    sg = gonioref.single_geometries[frame]
    sg.geometry_refinement.set_param(gonioref.get_ai(sg.get_position()).param)
    sg.geometry_refinement.refine3(fix=["wavelength"])
    angles.append(numpy.rad2deg(get_pos(frame)))
    dist.append(gonioref.single_geometries[frame].geometry_refinement.dist)
    poni1.append(gonioref.single_geometries[frame].geometry_refinement.poni1)
    poni2.append(gonioref.single_geometries[frame].geometry_refinement.poni2)
    rot1.append(gonioref.single_geometries[frame].geometry_refinement.rot1)
    rot2.append(gonioref.single_geometries[frame].geometry_refinement.rot2)
```

```
Wall time: 6.92 s
```

In [20]:

```
fig,ax = subplots(5, figsize=(9,2*5))
angles = numpy.array(angles)
dist = numpy.array(dist)
poni1 = numpy.array(poni1)
poni2 = numpy.array(poni2)
rot1 = numpy.array(rot1)
rot2 = numpy.array(rot2)

ax[0].plot(angles, dist)
ax[1].plot(angles, poni1)
ax[2].plot(angles, poni2)
ax[3].plot(angles, numpy.rad2deg(rot1))
ax[4].plot(angles, numpy.rad2deg(rot2))


ax[0].set_ylabel("Distance (m)")
ax[1].set_ylabel("Poni1 (m)")
ax[2].set_ylabel("Poni2 (m)")
ax[3].set_ylabel("Rot1 (°)")
ax[4].set_ylabel("Rot2 (°)")


ax[4].set_xlabel("Azimuth (°)")

plt.tight_layout()
```

Figure

In [41]:

```
def re_extract(idx):
    frame = frames[idx]
    sg = gonioref.single_geometries[frame]
    sg.geometry_refinement.set_param(gonioref.get_ai(sg.get_position()).param)
    sg.extract_cp(Imin=sg.image.mean())

#re_extract(37)
#re_extract(83)
#re_extract(277)
#re_extract(299)
#re_extract(303)
#re_extract(354)
#re_extract(80)
# on a second pass
if True:
    for idx in range(0,360):
        re_extract(idx)

gonioref.refine3()
geometries = copy.copy(gonioref.single_geometries)
```

```
Free parameters: ['dist', 'poni1', 'poni2', 'rot1', 'rot2', 'rot_x', 'rot_y']
Fixed: {}
     fun: 1.5909739379404823e-09
     jac: array([-1.25917675e-07,  1.41314517e-08,  6.36925502e-07, -4.99286640e-07,
        6.52494255e-08,  2.26607245e-08,  1.46298260e-07])
 message: 'Optimization terminated successfully'
    nfev: 16
     nit: 2
    njev: 2
  status: 0
 success: True
       x: array([ 3.49588129e+00,  1.82398057e-02,  8.54554509e-01, -3.97675960e-03,
        2.69594081e-03, -9.57078157e-03, -1.39570667e-02])
Constrained Least square 1.6134862722394653e-09 --> 1.5909739379404823e-09
maxdelta on rot1: -0.003970167501749038 --> -0.0039767595975896235
```

In [42]:

```
%%time
fraction = 1
angles = []
dist = []
poni1 = []
poni2 = []
rot1 = []
rot2 = []
for idx in range(0,len(frames),fraction):
    frame = frames[idx]
    sg = gonioref.single_geometries[frame]
    sg.geometry_refinement.set_param(gonioref.get_ai(sg.get_position()).param)
    sg.geometry_refinement.refine3(fix=["wavelength"])
    angles.append(numpy.rad2deg(get_pos(frame)))
    dist.append(gonioref.single_geometries[frame].geometry_refinement.dist)
    poni1.append(gonioref.single_geometries[frame].geometry_refinement.poni1)
    poni2.append(gonioref.single_geometries[frame].geometry_refinement.poni2)
    rot1.append(gonioref.single_geometries[frame].geometry_refinement.rot1)
    rot2.append(gonioref.single_geometries[frame].geometry_refinement.rot2)
```

```
Wall time: 1min 43s
```

In [43]:

```
fig,ax = subplots(5, figsize=(9,2*5))
angles = numpy.array(angles)
dist = numpy.array(dist)
poni1 = numpy.array(poni1)
poni2 = numpy.array(poni2)
rot1 = numpy.array(rot1)
rot2 = numpy.array(rot2)

ax[0].plot(angles, dist)
ax[1].plot(angles, poni1)
ax[2].plot(angles, poni2)
ax[3].plot(angles, numpy.rad2deg(rot1))
ax[4].plot(angles, numpy.rad2deg(rot2))

ax[0].set_ylabel("Distance (m)")
ax[1].set_ylabel("Poni1 (m)")
ax[2].set_ylabel("Poni2 (m)")
ax[3].set_ylabel("Rot1 (°)")
ax[4].set_ylabel("Rot2 (°)")
ax[4].set_xlabel("Rot3 (°)")
plt.tight_layout()
```

Figure

In [44]:

```
gonioref.bounds= {
                }
gonioref.refine3(method="simplex")
```

```
WARNING:pyFAI.goniometer:No bounds for optimization method Nelder-Mead
```

```
Free parameters: ['dist', 'poni1', 'poni2', 'rot1', 'rot2', 'rot_x', 'rot_y']
Fixed: {}
 final_simplex: (array([[ 3.49594603e+00,  1.80531220e-02,  8.54561673e-01,
        -4.03438367e-03,  2.77430577e-03, -9.85306375e-03,
        -1.41662706e-02],
       [ 3.49594603e+00,  1.80531220e-02,  8.54561673e-01,
        -4.03438367e-03,  2.77430577e-03, -9.85306375e-03,
        -1.41662706e-02],
       [ 3.49594603e+00,  1.80531220e-02,  8.54561673e-01,
        -4.03438367e-03,  2.77430577e-03, -9.85306375e-03,
        -1.41662706e-02],
       [ 3.49594603e+00,  1.80531220e-02,  8.54561673e-01,
        -4.03438367e-03,  2.77430577e-03, -9.85306375e-03,
        -1.41662706e-02],
       [ 3.49594603e+00,  1.80531220e-02,  8.54561673e-01,
        -4.03438367e-03,  2.77430577e-03, -9.85306375e-03,
        -1.41662706e-02],
       [ 3.49594603e+00,  1.80531220e-02,  8.54561673e-01,
        -4.03438367e-03,  2.77430577e-03, -9.85306375e-03,
        -1.41662706e-02],
       [ 3.49594603e+00,  1.80531220e-02,  8.54561673e-01,
        -4.03438367e-03,  2.77430577e-03, -9.85306375e-03,
        -1.41662706e-02],
       [ 3.49594603e+00,  1.80531220e-02,  8.54561673e-01,
        -4.03438367e-03,  2.77430577e-03, -9.85306375e-03,
        -1.41662706e-02]]), array([1.58636671e-09, 1.58636671e-09, 1.58636671e-09, 1.58636671e-09,
       1.58636671e-09, 1.58636671e-09, 1.58636671e-09, 1.58636671e-09]))
           fun: 1.5863667127927179e-09
       message: 'Optimization terminated successfully.'
          nfev: 1178
           nit: 706
        status: 0
       success: True
             x: array([ 3.49594603e+00,  1.80531220e-02,  8.54561673e-01, -4.03438367e-03,
        2.77430577e-03, -9.85306375e-03, -1.41662706e-02])
Constrained Least square 1.5909739379404823e-09 --> 1.5863667127927179e-09
maxdelta on rot_x: -0.009570781574584322 --> -0.00985306375499262
```

Out[44]:

```
1.5863667127927179e-09
```

In [45]:

```
for k,v in zip(gonioref.nt_param._fields, gonioref.param):
    print(f"{k:>12s}: {v}")
```

```
        dist: 3.4959460321091145
       poni1: 0.01805312198518727
       poni2: 0.8545616732718262
        rot1: -0.004034383668064181
        rot2: 0.0027743057744187257
       rot_x: -0.00985306375499262
       rot_y: -0.014166270645843956
```

In [46]:

```
gonioref.save("calibration_output.json")
```

In [47]:

```
if True: #Create a MultiGeometry integrator from the refined geometry:

    gonioref.sload('calibration_output.json')

    angles = []
    images = []
    for sg in gonioref.single_geometries.values():
        angles.append(sg.get_position())
        images.append(sg.image)
    
    multigeo = gonioref.get_mg(angles)
    multigeo.radial_range=(0, 15)
    multigeo.azimuth_range=(-180, 180)
    print(multigeo)


    %time res = multigeo.integrate2d(images, npt_rad=5000, npt_azim=360, method=("no", "histogram", "cython"))
    
    jupyter.plot2d(res)
    
    if False: #W 2D image
        fabio.edfimage.EdfImage(data=res.intensity).save("regrouped.edf")
        fabio.tifimage.TifImage(data=res.intensity).save("regrouped.tif")
```

```
MultiGeometry integrator with 360 geometries on (0, 15) radial range (2th_deg) and (-180, 180) azimuthal range (deg)
Wall time: 1min 49s
```

Figure

In [48]:

```
if True: # Calculate the optimal number of points for integration
    over = 3
    npt = int(over * numpy.deg2rad(max(multigeo.radial_range) - min(multigeo.radial_range)) /
          numpy.arctan2(pilatus.pixel1, gonioref.nt_param(*gonioref.param).dist))
    print("Number of bins: %s"%npt)
    
    %time res2 = multigeo.integrate1d(images, npt)
    jupyter.plot1d(res2)
    pass
    
    if True:
        numpy.savetxt(f"1D_integration.x_y", numpy.array((res2.radial, res2.intensity)).T)
```

```
Number of bins: 15963
Wall time: 3min 12s
```

Figure

In [49]:

```
if True:   #needs res2 from 1D integration, above

    # Line profile function...
    #Peak profile

    from scipy.interpolate import interp1d
    from scipy.optimize import bisect

    def calc_fwhm(integrate_result, calibrant):
        "calculate the tth position and FWHM for each peak"
        delta = integrate_result.intensity[1:] - integrate_result.intensity[:-1]
        maxima = numpy.where(numpy.logical_and(delta[:-1]>0, delta[1:]<0))[0]
        minima = numpy.where(numpy.logical_and(delta[:-1]<0, delta[1:]>0))[0]
        maxima += 1
        minima += 1
        tth = []
        FWHM = []
        for tth_rad in calibrant.get_2th():
            tth_deg = tth_rad*integrate_result.unit.scale
            if (tth_deg<=integrate_result.radial[0]) or (tth_deg>=integrate_result.radial[-1]):
                continue
            idx_theo = abs(integrate_result.radial-tth_deg).argmin()
            id0_max = abs(maxima-idx_theo).argmin()
            id0_min = abs(minima-idx_theo).argmin()
            I_max = integrate_result.intensity[maxima[id0_max]]
            I_min = integrate_result.intensity[minima[id0_min]]
            tth_maxi = integrate_result.radial[maxima[id0_max]]
            I_thres = (I_max + I_min)/2.0
            if minima[id0_min]>maxima[id0_max]:
                if id0_min == 0:
                    min_lo = integrate_result.radial[0]
                else:
                    min_lo = integrate_result.radial[minima[id0_min-1]]
                min_hi = integrate_result.radial[minima[id0_min]]
            else:
                if id0_min == len(minima) -1:
                    min_hi = integrate_result.radial[-1]
                else:
                    min_hi = integrate_result.radial[minima[id0_min+1]]
                    min_lo = integrate_result.radial[minima[id0_min]]

            f = interp1d(integrate_result.radial, integrate_result.intensity-I_thres)
            tth_lo = bisect(f, min_lo, tth_maxi)
            tth_hi = bisect(f, tth_maxi, min_hi)
            FWHM.append(tth_hi-tth_lo)
            tth.append(tth_deg)
        return tth, FWHM

#____________________

    #Fit against Caglioti's formula:
    # FWHM^2 = Utan2 + Vtan + W
    tth_deg, FWHM_deg = calc_fwhm(res2, LaB6)

    def model_Caglioti(tth_deg, U, V, W):
        tantheta = numpy.tan(numpy.deg2rad(tth_deg)/2.0)
        FWHM2 = U*tantheta*tantheta + V*tantheta + W
        return numpy.rad2deg(numpy.sqrt(FWHM2))

#_____________________

    from scipy.optimize import curve_fit
    fit,cov = curve_fit(model_Caglioti, tth_deg, FWHM_deg, p0=[1e-6,1e-7,1e-8])
    print(fit)
    #print(cov)

    fig, ax = subplots(figsize=(10,8))
    #ax.plot(*calc_fwhm(res, LaB6), "o", label="rot2")
    ax.plot(*calc_fwhm(res2, LaB6), "o", label="full unconstrained refinement")
    # for lbl, sg in gonioref2d.single_geometries.items():
    #     ai = gonioref2d.get_ai(sg.get_position())
    #     img = sg.image * ai.dist * ai.dist / ai.pixel1 / ai.pixel2
    #     res = ai.integrate1d(img, 5000, unit="2th_deg", method="splitpixel")
    #     t,w = calc_fwhm(res, calibrant=calibrant)
    #     ax.plot(t, w,"-o", label=lbl)
    #ax.set_ylim(0.005, 0.010)
    ax.set_title("Peak profile as function of the angle")
    ax.set_ylabel("FWHM of peaks (in degrees)")
    ax.set_xlabel(res.unit.label)
    ax.plot(tth_deg, model_Caglioti(tth_deg, *fit), label="U:%.1e, V:%.1e, W:%.1e"%(fit[0], fit[1], fit[2]))
    ax.legend()
    pass
```

```
[ 7.44500012e-07 -1.45996093e-07  4.23362427e-08]
```

Figure

Produce the AI at static position (270 is the most commonly used)

In [50]:

```
integrate_me=gonioref.get_ai(numpy.deg2rad(270))
```

In [51]:

```
print(integrate_me)
integrate_me.save('calibration_output.poni')
```

```
Detector Pilatus CdTe 900kw	 PixelSize= 1.720e-04, 1.720e-04 m
Wavelength= 3.898874e-11 m
SampleDetDist= 3.495946e+00 m	PONI= 3.221939e-02, 8.447086e-01 m	rot1=-0.002774  rot2=-0.004034  rot3=4.712389 rad
DirectBeamDist= 3495.988 mm	Center: x=4967.485, y=105.321 pix	Tilt= 0.281° tiltPlanRotation= -55.485° 𝛌= 0.390Å
```

In [52]:

```
if True:
    with open("calibration_output.poni") as f:
        print(f.read())
```

```
# Nota: C-Order, 1 refers to the Y axis, 2 to the X axis 
# Calibration done at Fri May 19 14:44:42 2023
poni_version: 2
Detector: PilatusCdTe900kw
Detector_config: {}
Distance: 3.4958811333382327
Poni1: 0.03219871267383986
Poni2: 0.8449842238674947
Rot1: -0.002695039198537887
Rot2: -0.003970167501749038
Rot3: 4.71238898038469
Wavelength: 3.898874164566046e-11
# Nota: C-Order, 1 refers to the Y axis, 2 to the X axis 
# Calibration done at Fri May 19 14:57:33 2023
poni_version: 2
Detector: PilatusCdTe900kw
Detector_config: {}
Distance: 3.4958811333382327
Poni1: 0.03219871267383986
Poni2: 0.8449842238674947
Rot1: -0.002695039198537887
Rot2: -0.003970167501749038
Rot3: 4.71238898038469
Wavelength: 3.898874164566046e-11
# Nota: C-Order, 1 refers to the Y axis, 2 to the X axis 
# Calibration done at Fri May 19 15:09:08 2023
poni_version: 2
Detector: PilatusCdTe900kw
Detector_config: {}
Distance: 3.4959460321091145
Poni1: 0.03221939263103123
Poni2: 0.8447086095168336
Rot1: -0.002774305774418726
Rot2: -0.004034383668064181
Rot3: 4.71238898038469
Wavelength: 3.898874164566046e-11
```

In [53]:

```
# calculate ai per azimuth

gonioref.sload("calibration_output.JSON")


angles_c = []
dist_c = []
poni1_c = []
poni2_c = []
rot1_c = []
rot2_c = []
for idx in range(0,360,):
    
    ai= gonioref.get_ai(numpy.deg2rad(idx+0.5))
    angles_c.append(idx+0.5)
    dist_c.append(ai.dist)
    poni1_c.append(ai.poni1)
    poni2_c.append(ai.poni2)
    rot1_c.append(ai.rot1)
    rot2_c.append(ai.rot2)
```

In [54]:

```
if True: # display fitted and calc ai

    fig,ax = subplots(5, figsize=(9,2*5))
    angles_c = numpy.array((angles_c))
    dist_c = numpy.array(dist_c)
    poni1_c = numpy.array(poni1_c)
    poni2_c = numpy.array(poni2_c)
    rot1_c = numpy.array(rot1_c)
    rot2_c = numpy.array(rot2_c)

    ax[0].plot(angles_c, dist_c, 'r-')
    ax[1].plot(angles_c, poni1_c, 'r-')
    ax[2].plot(angles_c, poni2_c,'r-')
    ax[3].plot(angles_c, numpy.rad2deg(rot1_c), 'r-')
    ax[4].plot(angles_c, numpy.rad2deg(rot2_c), 'r-')

    angle_o = numpy.array(numpy.rad2deg(angles))
    dist = numpy.array(dist)
    poni1 = numpy.array(poni1)
    poni2 = numpy.array(poni2)
    rot1 = numpy.array(rot1)
    rot2 = numpy.array(rot2)

    ax[0].plot(angle_o, dist)
    ax[1].plot(angle_o, poni1)
    ax[2].plot(angle_o, poni2)
    ax[3].plot(angle_o, numpy.rad2deg(rot1))
    ax[4].plot(angle_o, numpy.rad2deg(rot2))

    ax[0].set_ylabel("Distance (m)")
    ax[1].set_ylabel("Poni1 (m)")
    ax[2].set_ylabel("Poni2 (m)")
    ax[3].set_ylabel("Rot1 (°)")
    ax[4].set_ylabel("Rot2 (°)")
    ax[4].set_xlabel("Rot3 (°)")
    plt.tight_layout()
```

Figure

In [57]:

```
if True: #series of 1D plots w/out W    
    plt.figure()
    plt.plot(res.intensity[::1].T)
    plt.show()


    rays = res.intensity[::1]
    print(rays.shape)

    for i, ray in enumerate(rays):
        #numpy.savetxt(f"ray_{i:03d}.txt", numpy.array((res.radial, ray)).T, header="radial intensity")
        numpy.savetxt(f"ray_{i:03d}.x_y", numpy.array((res.radial, ray)).T)
```

Figure

```
(360, 5000)
```

In [58]:

```
# image is rotated


if True:
    import polarTransform   # uses: https://github.com/addisonElliott/polarTransform
    import matplotlib.pyplot as plt

    polarimage=(res.intensity)

    cartesianImage,ptSettings = polarTransform.convertToCartesianImage(polarimage,
                                                                       initialAngle=0 *numpy.pi,
                                                                       finalAngle=2 * numpy.pi)
    plt.figure()
    plt.subplot(211)
    plt.imshow(cartesianImage, cmap='hot', vmin=1e9, vmax=1e12)
    plt.subplot(212)
    plt.imshow(polarimage, cmap='hot', vmin=1e9, vmax=1e12)
    
    if True:
        fabio.tifimage.TifImage(cartesianImage).save("cartesianImage.tif")
```

Figure

In [ ]:

```

```
